# Full-length transcriptomic identification of R2R3-MYB family genes related to secondary cell wall development in *Cunninghamia lanceolata* (Chinese fir)

**DOI:** 10.1186/s12870-021-03322-w

**Published:** 2021-12-08

**Authors:** Hebi Zhuang, Sun-Li Chong, Borah Priyanka, Xiao Han, Erpei Lin, Zaikang Tong, Huahong Huang

**Affiliations:** State Key Laboratory of Subtropical Silviculture, Zhejiang A&F University, Lin’an, Hangzhou, 311300 China

**Keywords:** Wood formation, MYB transcription factor, Gymnosperm, *Cunninghamia lanceolata*, Evolutionary relationship

## Abstract

**Background:**

R2R3-MYB is a class of transcription factor crucial in regulating secondary cell wall development during wood formation. The regulation of wood formation in gymnosperm has been understudied due to its large genome size. Using Single-Molecule Real-Time sequencing, we obtained full-length transcriptomic libraries from the developmental stem of *Cunninghamia lanceolata*, a perennial conifer known as Chinese fir. The R2R3-MYB of *C. lanceolata* (hereafter named as ClMYB) associated with secondary wall development were identified based on phylogenetic analysis, expression studies and functional study on transgenic line.

**Results:**

The evolutionary relationship of 52 ClMYBs with those from *Arabidopsis thaliana*, *Eucalyptus grandis*, *Populus trichocarpa*, *Oryza sativa*, two gymnosperm species, *Pinus taeda*, and *Picea glauca* were established by neighbour-joining phylogenetic analysis. A large number of ClMYBs resided in the woody-expanded subgroups that predominated with the members from woody dicots. In contrast, the woody-preferential subgroup strictly carrying the members of woody dicots contained only one candidate. The results suggest that the woody-expanded subgroup emerges before the gymnosperm/angiosperm split, while most of the woody-preferential subgroups are likely lineage-specific to woody dicots. Nine candidates shared the same subgroups with the *A. thaliana* orthologs, with known function in regulating secondary wall development. Gene expression analysis inferred that *ClMYB1/2/3*/4/5/*26/27/49/51* might participate in secondary wall development, among which *ClMYB1/2/5/26/27/49* were significantly upregulated in the highly lignified compression wood region, reinforcing their regulatory role associated with secondary wall development. ClMYB1 was experimentally proven a transcriptional activator that localised in the nucleus. The overexpression of ClMYB1 in *Nicotiana benthamiana* resulted in an increased lignin deposition in the stems. The members of subgroup S4, ClMYB3/4/5 shared the ERF-associated amphiphilic repression motif with AtMYB4, which is known to repress the metabolism of phenylpropanoid derived compounds. They also carried a core motif specific to gymnosperm lineage, suggesting divergence of the regulatory process compared to the angiosperms.

**Conclusions:**

This work will enrich the collection of full-length gymnosperm-specific R2R3-MYBs related to stem development and contribute to understanding their evolutionary relationship with angiosperm species.

**Supplementary Information:**

The online version contains supplementary material available at 10.1186/s12870-021-03322-w.

## Background

The radial growth of woody stem in gymnosperm and angiosperm is contributed by bifacial development of cambium cells that forms xylem and phloem to the inner and outer part of vascular bundles, respectively [1]. Even though the xylems subsequently differentiate into vessels and fibres in angiosperms, gymnosperms form a primitive lineage having tracheids in woody tissues for water conductance and mechanical support. In a matured xylem cell, the secondary cell wall forms the thickest layer that determines wood properties. The secondary cell wall is formed by a structural network consisting of cellulose as load-bearing support infiltrated by lignin and hemicellulose to reinforce wall support. To maintain sustainable growth, woody plants possess tight regulation on cambial activity and its wood formation incoherent with developmental phases and response to environmental stimuli [2]. Cell wall formation is spatiotemporally controlled by a hierarchical regulatory network. Transcription factors containing NAC (NAM/ATAF/CUC) [3–5] and MYB domains [6, 7] are essential for the transcriptional control of wood formation.

The MYB proteins constitute one of the largest transcription factor families in plants. All members have a highly conserved DNA-binding domain, i.e., the domain with one to three imperfect amino acid sequence repeats (R1, R2 and R3). They can be categorized according to the number of adjacent R proteins as R1, R2R3, 3R or 4R proteins with one, two, three or four R proteins, respectively [8]. The R2R3-MYB subfamily has the highest number of MYB members. Some R2R3-MYB members have crucial roles in regulating secondary cell wall formation [9, 10]. In *Arabidopsis thaliana*, the paralogous genes *AtMYB46* and *AtMYB83* are the second-level master switches that regulate secondary cell wall formation, and several other genes (*AtMYB4*, *AtMYB7*, *AtMYB20*, *AtMYB32*, *AtMYB42*, *AtMYB43*, *AtMYB52*, *AtMYB54*, *AtMYB58*, *AtMYB63*, *AtMYB69*, *AtMYB85*, and *AtMYB103*) may function as downstream transcription factors in this regulatory network [11]. Some R2R3-MYB members were involved in forming the secondary cell wall in tree species via the regulation of cellulose, hemicellulose, and lignin biosynthesis [12]. Like *AtMYB46* and *AtMYB83*, *PtrMYB3* and *PtrMYB20* from *Populus trichocarpa* are also second-level master switches involved in the regulation of secondary cell wall formation, which induce the expression of cellulose, xylan, and lignin biosynthesis genes [13]. In addition, *EgrMYB1*, *EgrMYB2*, and *EgrMYB88* of *Eucalyptus grandis* also involve in the regulation of secondary cell wall formation [14–16]. In conifers, *PtMYB1*, *PtMYB4*, and *PtMYB8* of *Pinus taeda* [17–19] have been suggested to regulate lignin biosynthesis in the differentiated xylem tissues.

Following land plants’ territorialisation, R2R3-MYBs have undergone clade-asymmetric expansion and subsequent sub- or neofunctionalization correspond with species diversification [20]. The genome-wide duplication and tandem duplication events contribute to the lineage-specific expansion [21]. A genome-wide analysis of R2R3-MYBs in various woody dicots such as *Eucalyptus grandis, P. trichocarpa and Vitis vinifera* has identified specific lineage expansion as woody-preferential and woody-expanded subgroups. These are implicated for essential functions at regulating wood formation [12]. The formation of tracheid in gymnosperm is regulated by the VNS-MYB based transcriptional network [22], reflecting the conserved function of MYB regulators to some extent among vascular plants. The evolutionary relationship of R2R3-MYBs between gymnosperm and angiosperm species remains elusive.

*Cunninghamia lanceolata* (Chinese fir), an evergreen conifer belonging to the Cupressaceae family, is native to China and distributed mainly in 16 provinces in southern China. Due to its rapid growth, desirable wood quality and low disease and insect pest pressure, this species has been widely cultivated for over 3000 years. Breeding efforts beginning in the 1960s, including provenance tests, cross-breeding and clonal selection aimed for rapid growth, have achieved remarkable successes. As a result, they currently account for 20–30% of China’s total commercial timber production. Recently, exploration of the molecular mechanism of wood formation in *C. lanceolata* was attempted by constructing a cDNA library and expressed sequence tag (EST) database of *C. lanceolata* var. Dugaushau [23] and a transcriptome database containing more than 80,000 sequences established using Illumina high-throughput sequencing [24]. Additionally, candidate genes related to lignin and cellulose biosynthesis were analysed in Wang et al. (2011) [25]. Despite that, identification and characterization of gene functions in *C. lanceolate* have been complicated by incomplete and redundant sequences obtained from Illumina short-read sequencing analysis. We used the PacBio long-read sequencing platform to obtain full-length transcriptomic libraries from the developmental stem of *C. lanceolata*. As such, the R2R3-MYBs of *C. lanceolata* (hereafter named as ClMYBs) were identified. Protein sequence characteristics, phylogenetic relationships, and expression patterns of ClMYB encoding genes were subsequently investigated. Based on their evolutionary relationship with the known genes from *Arabidopsis thaliana* and woody species, we identified nine ClMYBs associated with secondary cell wall development with some degree of diversification of tissue specificity compared to the angiosperm species. The involvement of *ClMYB1* in secondary wall development was identified and characterized by ectopic expression in tobacco. These results provide essential genetic resources for improving the wood quality of *C. lanceolata* at the molecular level.

## Materials and methods

### Plant materials

The plant materials were harvested from two-year-old *C.lanceolata* clone 2015–2, obtained from the Forest Germplasm Resource Conservation Nursery (E119.726, N30.267) located at the State Key Laboratory of Subtropical Silviculture, Zhejiang A&F University. The nursery was maintained by our laboratory; hence no permission for the sample collection was required. To our knowledge there was no herbarium available for the deposition of the voucher specimen of this specific plant. Plant identification was carried out by the co-author, Professor Zaikang Tong. Plants were grown at 26-28 °C under a light intensity of 1500 Lux and a photoperiod of 12 h daily. Eleven different plant organs and tissues were collected from mid-March until the end of June in 2017. Among these organs, female cones (FC) and male cones (MC) were harvested from upper branches at similar heights. The roots (RT) were cut from hydroponically grown clone 2015–2 plants. Leaves (L) consisted of the one-year-old mature coniferous needles. Five stem segments were collected from the top to bottom of an ongoing-year branch, with each segment being 2 cm long and marked sequentially as S1, S2, S3, S4, and S5. Phloroglucinol-HCl staining was performed on the sequential stem cross-sections to observe vascular development (Supplementary Fig. S1). The bark (B) of a two-year-old stem segment was collected and the 1.0-mm-thick xylem (X) layer was scraped from the outer xylem. All samples were quickly frozen in liquid nitrogen and stored at − 80 °C. *Nicotiana benthamiana* was used for genetic transformation for subsequent analysis of the gene functions.

### Compression wood induction

An artificial bending treatment was performed to induce compression wood [26]. In early May of 2017, stem of *C. lanceolata* plants that displayed relatively uniform growth were bent at an angle of approximately 45° to the vertical direction of the ground. The trunk segments at the bending site were sampled after 10, 30 and 60 days of bending. After the bark was removed, the outer xylem was scraped from the compression wood (CW) and opposite wood (OW) and was quickly frozen in liquid nitrogen. Untreated, upright plants were used as controls (CK). Five biological replicates from the CW and OW samples developed at each time point and control plant at the zero-time point were harvested for the test.. Transverse sections (thickness of 10 μm) were prepared with the use of stem segments harvested after 60 days of bending. To visualize the thickening of the cell wall, the sections were stained with safranin. In addition, the double-wall thickness of tracheid was measured using a modification of Franklin’s method [27]. At least 90 tracheids from each wood type were measured using a light microscope, and the data were analysed for significant differences using SPSS 19.0 (IBM) with Student’s t-test.

### Preparation of full-length transcriptomic library

The RNA from five stem segments were subjected to PacBio library construction and sequencing. The cDNAs were synthesized with Clontech SMARTer PCR cDNA Synthesis Kit according to the manufacturer’s protocol and the PCR products were sequenced with the PacBio Sequel System. The long-read sequence was analysed by Single-Molecule Real-Time (SMRT) analysis software (v. 2.3.0) using the RS_IsoSeq.1 protocol (https://github.com/PacificBiosciences/). High-quality consensus isoforms were obtained based on the Iterative Clustering for Error Correction (ICE) algorithm and subsequent Quiver polishing. All these isoforms were further clustered and filtered using CD-Hit (v. 4.6) [28] to obtain unigenes.

### Identification of R2R3-MYB family members in *C. lanceolata*

The Hidden Markov Model (HMM) file for the MYB domain (PF00249) was downloaded from the Pfam database (http://pfam.sanger.ac.uk/). The putative MYB members obtained from the full-length transcriptome of *C. lanceolata* were identified by HMMER 3.0 [29] and the putative sequences containing MYB domain were identified. The protein sequences were analysed via SMART (http://smart.embl-heidelberg.de/) and InterPro (http://www.ebi.ac.uk/interpro/) to examine the topological arrangement of MYB domains in the sequences. BLASTp searches in conjunction with the nonredundant library (https://blast.ncbi.nlm.nih.gov/Blast.cgi) were used to aid in annotating the sequences containing R2R3-MYB domain. ClustalX multiple sequence alignment tool (v. 2.1) [30] was employed to filter redundant sequences of high similarity .

### Protein sequence analysis and phylogenetic tree construction

The ExPASy database (https://expasy.org) was used to analyse the physicochemical properties of R2R3-MYBs in *C. lanceolata*. Multiple sequence alignments were performed using ClustalX, and sequence logos of the R2 and R3 domains were plotted via the WebLogo (http://weblogo.Berkeley.edu/logo.cgi). The motifs of the R2R3-MYBs in *C. lanceolata* were analysed using the MEME program (http://meme-suite.org/tools/meme). The motif width was set to 6–50, and the number of motifs was 20, with the default values for the remaining parameters.

The evolutionary relationship of *C. lanceolata* (52) R2R3-MYBs with *E. grandis* (141), *P. trichocarpa* (187), *A. thaliana* (126), *Oryza sativa* (104), *P. taeda* (7), and *Picea glauca* (12) were studied via the construction of a Neighbour-Joining phylogenetic tree with 1000 bootstrap replicates. The sequences of the species other than *C. lanceolata* were downloaded from the Phytozome (http://www.phytozome.org/) and NCBI (http://www.ncbi.nlm.nih.gov/) databases. A total of 629 R2R3-MYBs sequences were aligned using ClustalW and the phylogenetic tree were constructed using MEGA7 [31]. The evolutionary distances were computed using the Jones-Taylor-Thornton matrix-based method and the rate variation among sites was modelled with a gamma distribution (shape parameter = 1).

### Expression profile analysis of *ClMYB*s based on RNA sequencing (RNA-Seq) and quantitative real-time PCR (qRT-PCR)

The RNA of female cone (FC), male cone (MC), needle-leaves (L), roots (RT), five stem segments (named as S1to S5), bark (B) and xylem (X) were used to construct RNA-Seq libraries with three biological replicates were included. Total RNA from *C. lanceolata* samples was extracted according to the PureLink Plant RNA Reagent Kit manual (ThermoFisher Scientific, USA). Paired-end 150-bp sequencing was performed on the Illumina HiSeq Xten platform. The transcript abundance of the *ClMYB*s in the different organs and tissues was reported in terms of fragments per kilobase of exon model per million mapped reads (FPKM). After they were log-transformed, the expression data were subjected to hierarchical clustering, and an expression profile heatmap was constructed by TBtools software [32].

The qRT-PCR analysis was performed using a PrimeScript Reagent Kit in conjunction with a SYBR Premix Ex Taq Kit (Takara, Japan). The qRT-PCR system consisted of 1 μg total RNA, 5 μl of SYBR Premix Ex Taq (2×), 0.2 μl of forward primer (10 μM), 0.2 μl of reverse primer (10 μM), 0.8 μl of the first-strand cDNA, and enough double-distilled water (ddH_2_O) to bring the total volume of 10 μl. The reaction was performed with the following the program: 95 °C for 2 min; 40 cycles of 95 °C for 5 s, and 60 °C for 30 s. The data obtained were analyzed by the 2-ΔΔCt method, with the housekeeping gene *ClActin* used as an internal control. All calculations were performed via CFX96 Manager version 1.6 (Bio-Rad, USA) and Excel 2007 software. The qRT-PCR primer sequences are listed in Supplementary Table S1.

### Subcellular localization, transcriptional activation assay and ectopic expression analysis of *ClMYB1*

The open reading frame (ORF) of *ClMYB1* was amplified by PCR with gene-specific primers and then inserted into a pCAMBIA1302 vector by restriction enzyme ligation, yielding a *ClMYB1::sGFP* (synthetic green fluorescent protein) fusion expression cassette, which was transformed into onion epidermal cells via the particle bombardment method. The fluorescence signals were observed and imaged via a Zeiss LSM510 confocal microscope (Carl Zeiss, Oberkochen, Germany).

The ORF of *ClMYB1* without its termination codon was inserted into a pGBKT7 vector (Clontech, USA) harbouring GAL4-BD via Gateway technology. The recombinant plasmid was subsequently transformed into the yeast (*Saccharomyces cerevisiae*) strain Gold2. Positive clones were selected on SD/−Trp media, and the transformants were plated and cultured on SD/−Trp/−His/−Ade/X-a-gal media for transcriptional activation analysis.

*ClMYB1* was inserted into a pCAMBIA13011 vector downstream to the 35S promoter by restriction enzyme digestion and ligation. This *p35S::ClMYB1* expression cassette was transformed into *N. benthamiana* via the leaf disc method, mediated by *Agrobacterium tumefaciens* strain GV3101. Phenotypic analysis of two-month-old transgenic plants (T3) was performed. The DNA was extracted by the cetyl-trimethylammonium bromide (CTAB) method, and the transformation events of *ClMYB1* were analysed via PCR. Total RNA was extracted via a TRIzol kit (ThermoFisher Scientific, USA), and the expression of *ClMYB1* was measured via semiquantitative RT-PCR (semi-qRT-PCR) as described by [24]. The relevant primer sequences are listed in Supplementary Table S1. Phloroglucinol-HCl staining was applied to observe the lignin deposition in the stems, and the relative lignin content in the stems was determined according to the lignin-thioglycolic acid (LTGA) method [33].

## Results

### Identification and domain analysis of ClMYBs

A total of 116 candidate sequences with MYB domains were selected from the full-length transcriptomic library, including three 4R-MYB, seven 3R-MYB, 70 R2R3-MYB, and 36 1R-MYB proteins. After the removal of redundant sequences, we identified 52 R2R3-MYB with complete ORF, which were sequentially named as *ClMYB1–52* (Supplementary Table S2).

**Fig. 1:**
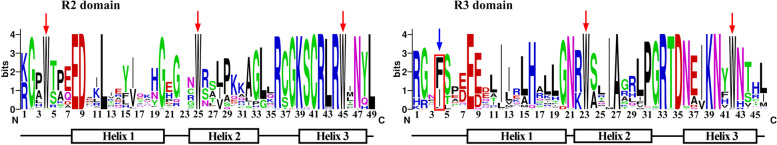
Sequence logo plots of R2 and R3 domains in 52 ClMYB proteins. Multiple alignments of 52 ClMYB domains was conducted by Clustal X. The bit score represented the information content for each amino acid residue. The three α-helices (Helix 1 to 3) position was marked with black boxes. Highly conserved tryptophan residues (W) were labelled with red arrows. The blue arrows indicated that the tryptophan residue was substituted by phenylalanine, isoleucine, or leucine

The number of amino acid residues in the 52 ClMYBs ranged from 215 to 596. The corresponding molecular weight and isoelectric points ranged between 24.3–64.1 kDa and 4.7–9.9 (Supplementary Table S2), respectively. Multiple sequence alignments showed that all the N-terminal regions of 52 ClMYBs contained conserved R2 and R3 domains. The R2 domains of ClMYBs contained three highly conserved Trp (W) residues. However, within the R3 domain, the first W residue is substituted by Leu (L), Ile (I) or Phe (F). In addition, some conserved amino acids were present around these W residues, including Gly (G2), Glu (E8), Asp (D9), Leu (L12), Gly (G20), Arg (R36), Gly (G38), Lys (K39), Cys (C41), Arg (R42), Arg (R44), Asn (N47) and Leu (L49) in the R2 domain; Glu (E8), His (H16), Gly (G20), Asn (N21), Gly (G32), Arg (R33), Thr (T34), Asp (D35), Asn (N36), Lys (K39) and Asn (N40) in the R3 domain (Fig. 1). These characteristics ensure that MYB domains fold into a stable helix-helix-turn-helix structure.

### A comparative phylogenetic study of R2R3-MYBs in *C. lanceolata* and other plant species

**Fig. 2:**
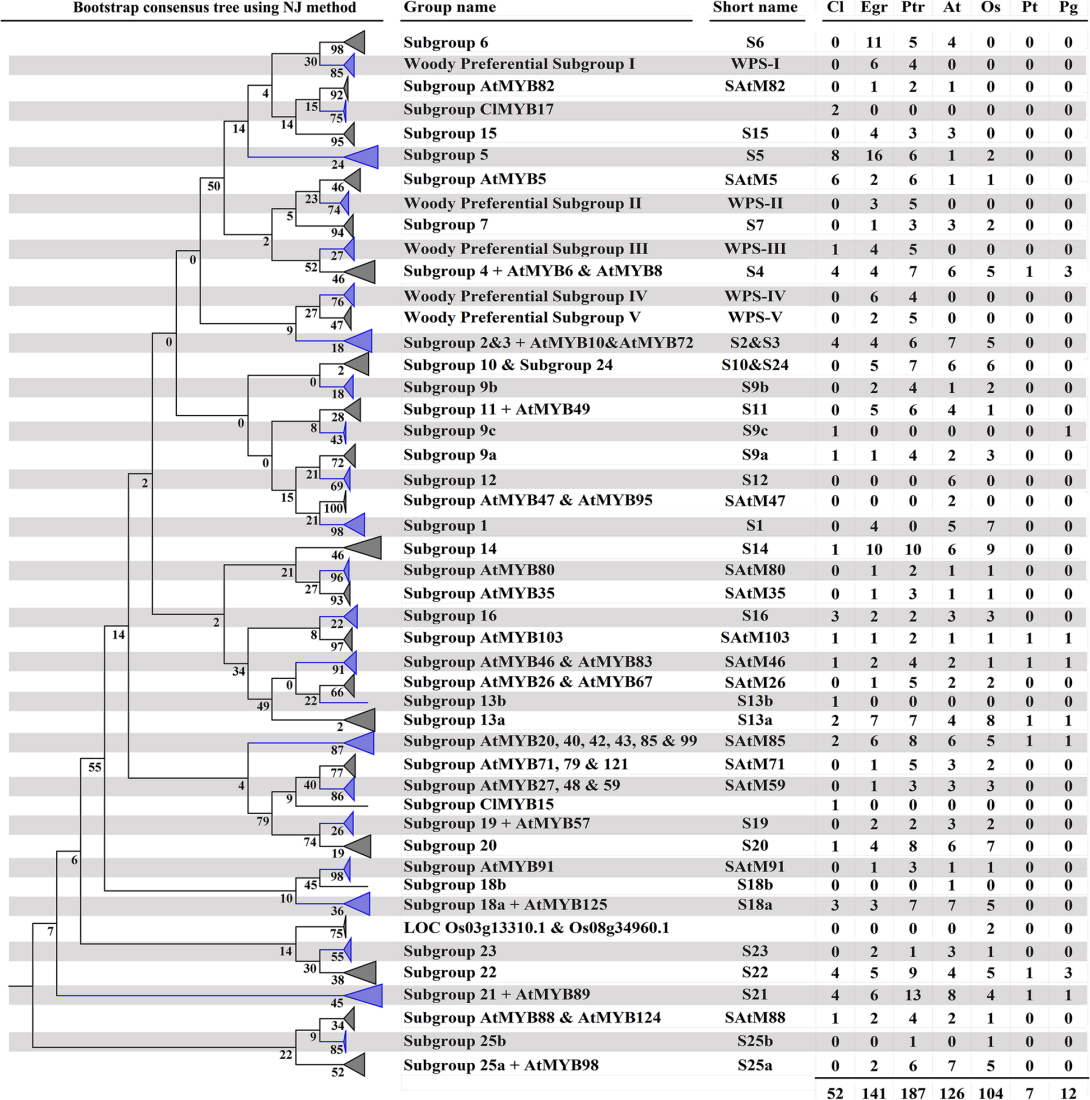
Phylogenetic relationship of R2R3-MYB proteins from *Cunninghamia lanceolata* (Cl), *Eucalyptus grandis* (Egr), *Populus trichocarpa* (Ptr), *Arabidopsis thaliana* (At), *Oryza sativa* (Os), *Pinus taeda* (Pt), and *Picea glauca* (Pg). All proteins were divided into 47 subgroups. The numbers of R2R3-MYB members in each subgroup in different species were listed on the right column

To analyse the evolutionary relationship of ClMYBs and known R2R3-MYBs from *A. thaliana, E. grandis, O. sativa, P. glauca*, *P. taeda and P. trichocarpa*, we constructed the neighbour-joining phylogenetic tree (Fig. 2; Supplementary Fig. S2). According to the topological structure, all the proteins could be divided into 47 subgroups, 21 of which harboured R2R3-MYBs from *C. lanceolata*. Over 50% of the subgroups were supported by bootstrap value of > 70%. Lower bootstrap values were found in the subgroups with mixed sequences of gymnosperm and angiosperm origin. This can be justified because many gene duplication events in the R2R3-MYBs occurred after divergence from a common ancestor [34] and rapid diversification of the family after the division [35]. Nomenclatures of the subgroups were previously reported for *A. thaliana* [8, 36], later these were modified by Soler et al., (2015) [12] following a genome-wide evolutionary analysis of the R2R3-MYBs from the woody species, *E. grandis*. The sequences of *A. thaliana* and *E. grandis* were correctly placed in the previously assigned subgroups. Three *C. lanceolata* specific subgroups were identified and they were named as subgroup 13b and ClMYB15, which harboured single candidate, ClMYB24 and ClMYB15, respectively. Subgroup ClMYB17 contained two candidates, ClMYB17 and ClMYB18. Furthermore, subgroup 9c containing ClMYB28 and PgMYB11 represented a gymnosperm specific lineage.

Only seven common subgroups (S4, SAtM103, SAtM46, S13a, SAtM85, S22, S21) contained sequences from all the selected plants (Fig. 2). For the MYBs that are important in regulating secondary wall development in *A. thaliana*, seventeen of them were distributed across nine subgroups (Table 1; Supplementary Fig. S2). AtMYB46 and AtMYB83 (subgroup SAtM46) were the second layer master switches to secondary wall developmental growth [37, 38]. AtMYB52, AtMYB54 and AtMYB69 (subgroup S21) were proposed to be the activator of lignin, xylan and cellulose biosynthesis [36, 39]. AtMYB20, AtMYB42, AtMYB43 and AtMYB85 (subgroup SAtM85) and AtMYB58 and AtMYB63 (subgroup S2 & S3) were the activators of lignin biosynthesis [40, 41]. AtMYB26 (subgroup SAtM26) was involved in secondary cell wall thickening in anther endothecium [42, 43]. AtMYB61 (subgroup SAtMYB61) was proposed to act as a mediator to allocate resources to sink tissues such as seed coat [44], root and xylem [45] by modulating genes related cell wall biosynthesis. Besides, two subgroups, namely S4 and S6, were dominated by MYBs functioning as antagonists of cell wall development. For example, AtMYB4 and AtMYB32 (subgroup S4) were the suppressors of biosynthesis of phenylpropanoid derived compounds [46, 47].

**Table 1:** Summary of R2R3-MYB transcription factors related to development of secondary cell wall

Function	R2R3-MYB related to SCW development	Group name	Homologous ***ClMYBs***	Biological process	Reference
Activator	AtMYB52, AtMYB54, AtMYB69	S21	ClMYB2	Lignin, xylan and cellulose biosynthesis	[36, 39]
	AtMYB20, AtMYB42, AtMYB43, AtMYB85 PtrMYB152 PtMYB1	SAtM85	ClMYB1 ClMYB51	Lignin biosynthesis. Binds to AtMYB4 to repress flavonoid biosynthesis	[19, 41, 48]
	AtMYB103 PtrMYB010, PtrMYB128	SAtM103	ClMYB26	Lignin biosynthesis	[5, 50]
	AtMYB46, AtMYB83 PtrMYB002, PtrMYB003, PtrMYB020, PtrMYB021 EgrMYB2 PtMYB4	SAtM46	ClMYB49	Lignin, xylan and cellulose biosynthesis	[14, 18, 37, 38, 51, 52]
	AtMYB26	SAtM26		Secondary wall thickening in anther endothecium	[42, 43]
	AtMYB58, AtMYB63	S2&S3		Lignin biosynthesis	[40]
	AtMYB61 PtMYB8	S13a	ClMYB27	Regulates stomatal aperture closure, seed coat mucilage biosynthesis, xylem and root development	[44, 45, 53]
Repressor	AtMYB4, AtMYB32 EgrMYB1	S4	ClMYB3, ClMYB4, ClMYB5	Biosynthesis of phenylpropanoid derived compound	[46, 47, 49]
	AtMYB75	S6		Anthocyanin biosynthesis	[54]

These subgroups implicated for secondary cell wall development in *A. thaliana* also included those from woody species such as seven MYBs in *P. trichocarpa* (PtrMYB152, PtrMYB010, PtrMYB128, PtrMYB002, PtrMYB003, PtrMYB020 and PtrMYB021) [5, 13, 48], two *E. grandis* proteins (EgrMYB1 and EgrMYB2) [14, 15, 49], and three *P. taeda* proteins (PtMYB1, PtMYB4, and PtMYB8) [18, 19] (Table 1; Supplementary Fig. S2). Not all ClMYBs were found in these secondary wall related subgroups. There were nine orthologous ClMYBs (ClMYB1, ClMYB2, ClMYB3, ClMYB4, ClMYB5, ClMYB26, ClMYB27, ClMYB49, and ClMYB51) segregated across six clades, suggesting they are the candidate regulators of secondary wall development during wood formation in *C. lanceolata* (Table 1). ClMYB49 belonging to subgroup SAtM46 was a homolog of AtMYB46 and AtMYB83, meanwhile ClMYB2 and ClMYB26 were clustered with subgroup S21 and SAtM103, respectively. For the subgroups associated with activation of lignin biosynthesis (subgroup SAtM85 and S2&S3), ClMYB1 and ClMYB51 belonged to SAtM85, while none were found in S2&S3. Meanwhile, ClMYB3, ClMYB4 and ClMYB5 were clustered with subgroup S4, which harbored the repressors of phenylpropanoid metabolism.

From the evolutionary analysis of R2R3-MYBs from *E. grandis*, MYBs of strictly woody species are detected in a total of five woody-preferential subgroups [12]. In the present study, we found that only ClMYB21 present in the woody-preferential subgroup III (Supplementary Fig. S2). Subgroup 5 (S5) and AtMYB5 (SAtM5) contained a high proportion of MYBs from woody perennial plant species and were referred to as woody-expanded subgroups, with eight and six ClMYBs assigned to these two subgroups, respectively.

### Motif analysis of R2R3-MYB proteins of *C. lanceolata*

**Fig. 3:**
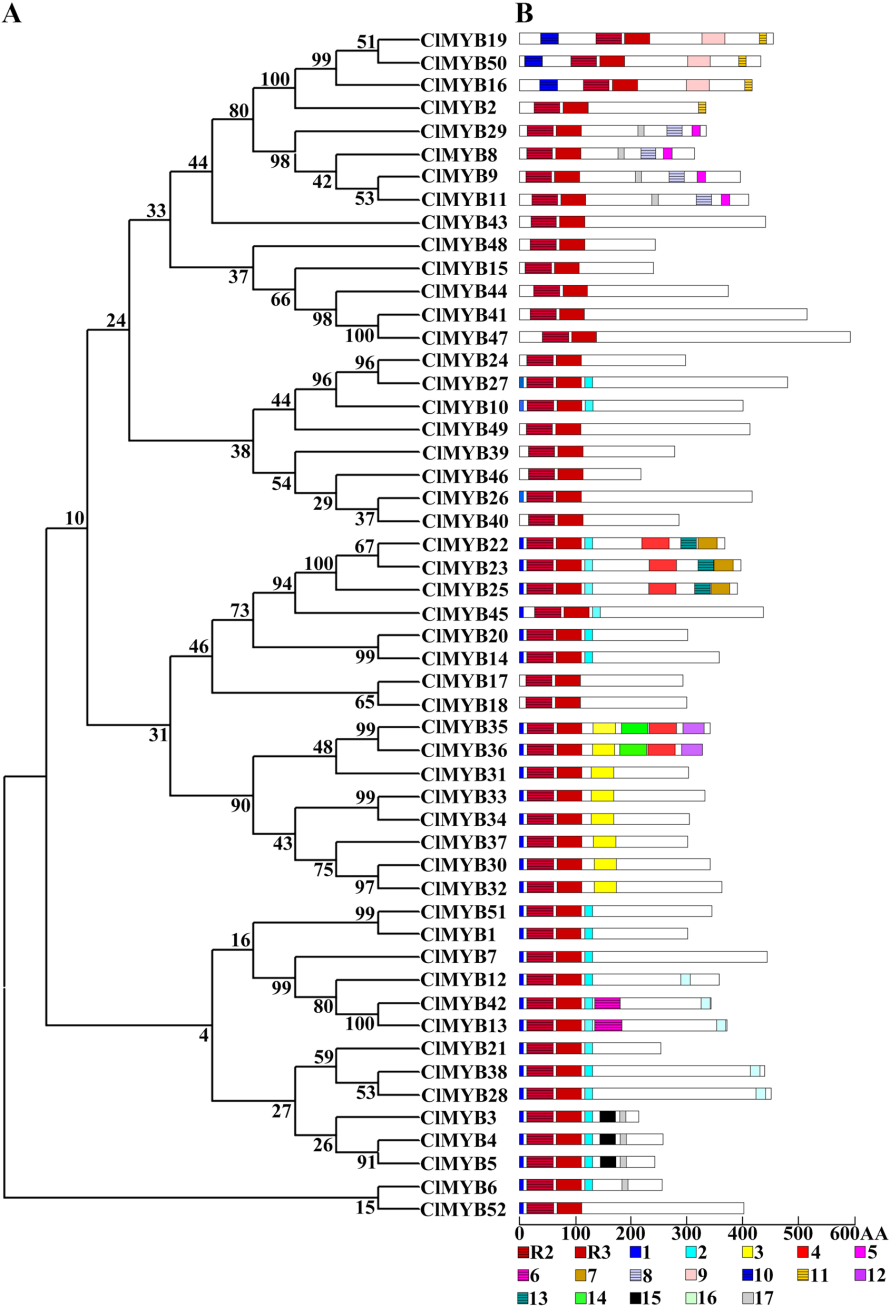
Phylogenetic relationships and conserved motifs distribution of ClMYB proteins. (**A**) Phylogenetic tree of 52 ClMYB transcription factors. (**B**) Schematic diagram of motifs distribution of ClMYB proteins. R2, R3, and the digits represented R2, R3 domains, and another 17 motifs, respectively

In addition to the R2 and R3 domains, 17 conserved motifs were identified via MEME; these motifs were designated as motifs 1 to 17 in increasing order of their corresponding E-values (Fig. 3; Supplementary Data S1). Motif 1 was upstream of the R2 domain, and the other motifs were downstream of the R3 domain (Fig. 3 B). Among the different subgroups, the amount and type of motifs were significantly different. Most of the members belonging to the same subgroup shared one or more motifs (Fig. 3A). For example, ClMYB8, ClMYB9, ClMYB11 and ClMYB29 belonging to S22 contained motif 5, motif 8, and motif 17. Motif 15 carrying the core sequence of (E/I)LIHMADFFQ was unique to ClMYB3, ClMYB4 and ClMYB5 in the subgroup S4. Whereas, motif 17 with the sequence of (D/E)(I/V)NLDL(C/S)ISLP was the ERF-associated amphiphilic repression (EAR) motif that common between angiosperm and gymnosperm in subgroup S4 and dictated the repressive role of the members on the phenylpropanoid metabolism [55].

### Expression profiles of *ClMYBs* in different organs and tissues

**Fig. 4:**
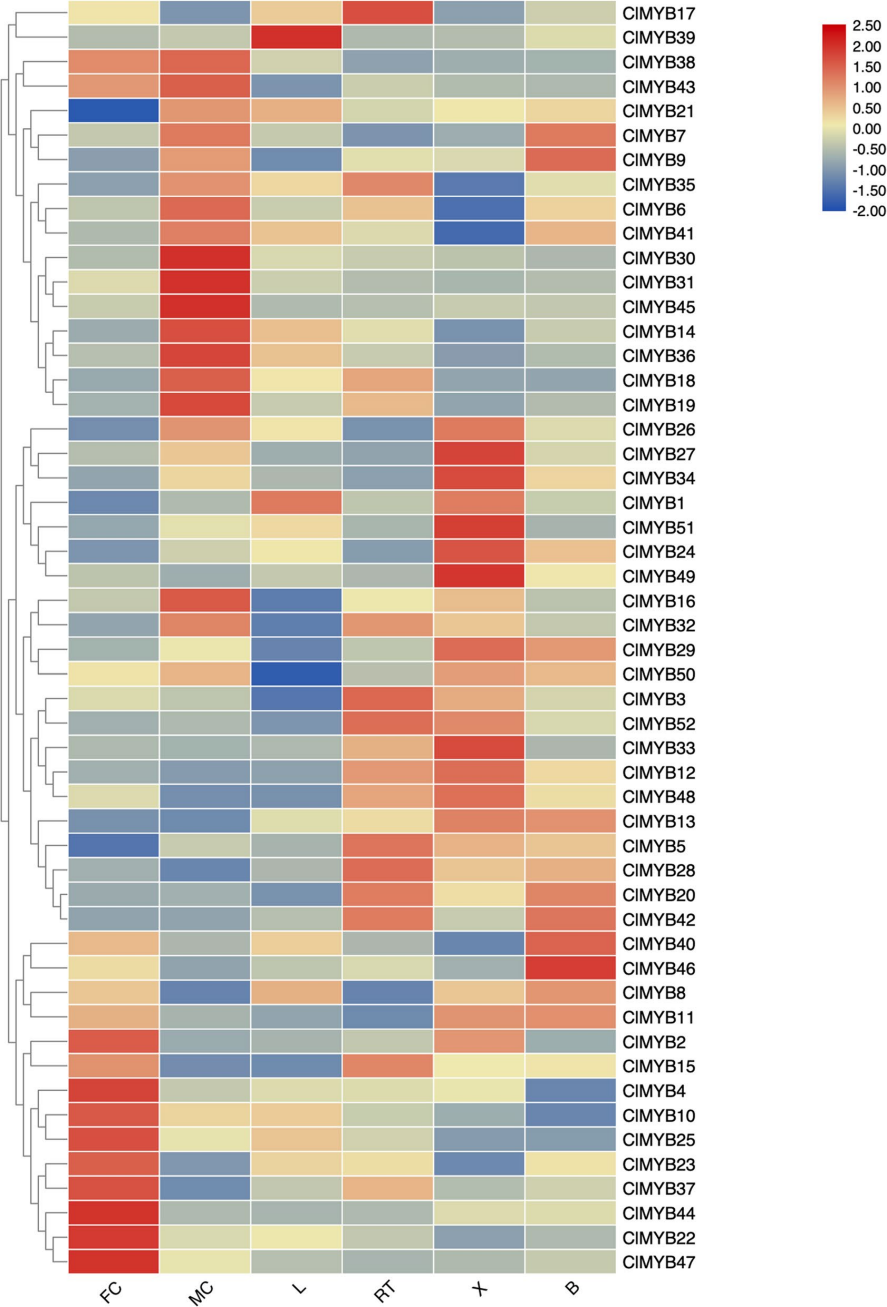
Expression profiles of *ClMYBs* in different tissues or organs. Log transformation and hierarchical clustering were performed for FPKM values of each gene. Log_2_(FPKM + 1) values were displayed according to the colour bar (at the bottom right corner). FC: female cones, MC: male cones, L: leaves, RT: roots, X: xylem, B: bark

To explore the involvement of ClMYB in the secondary wall development, expression profiles of the 52 ClMYB encoding genes in the six organs and tissues were analyzed using the available transcriptome data. Forty-three *ClMYB* were expressed in all the organs and tissues tested (FPKM> 0), eight of which (*ClMYB5*, *8*, *9*, *11*, *17*, *23*, *25*, and *47*) were constitutively expressed (FPKM> 2 in all samples) (Fig. 4; Supplementary Table S3). Compared with the other *ClMYBs*, *ClMYB9* showed relatively higher expression levels in the organs and tissues examined, with a corresponding mean FPKM value of 100.4. In some cases, the expression profiles of closely related members were similar. For example, the expression patterns of *ClMYB22*, *ClMYB23*, and *ClMYB25* in the subgroup SAtM5, one of the woody-expanded subgroups, they were clustered in two sub-clades that were highly expressed in female cone. However, in most cases, the expression profiles of the genes present in the same subgroup differed, suggesting their specific roles in different organs. This notion was manifested by *ClMYB14* and *ClMYB45*, though they also belonged to subgroup SAtM5, they were highly expressed in the male cone. Likewise, the expression of another SAtM5 ortholog, *ClMYB20* was highly expressed in root and bark tissues.

**Fig. 5:**
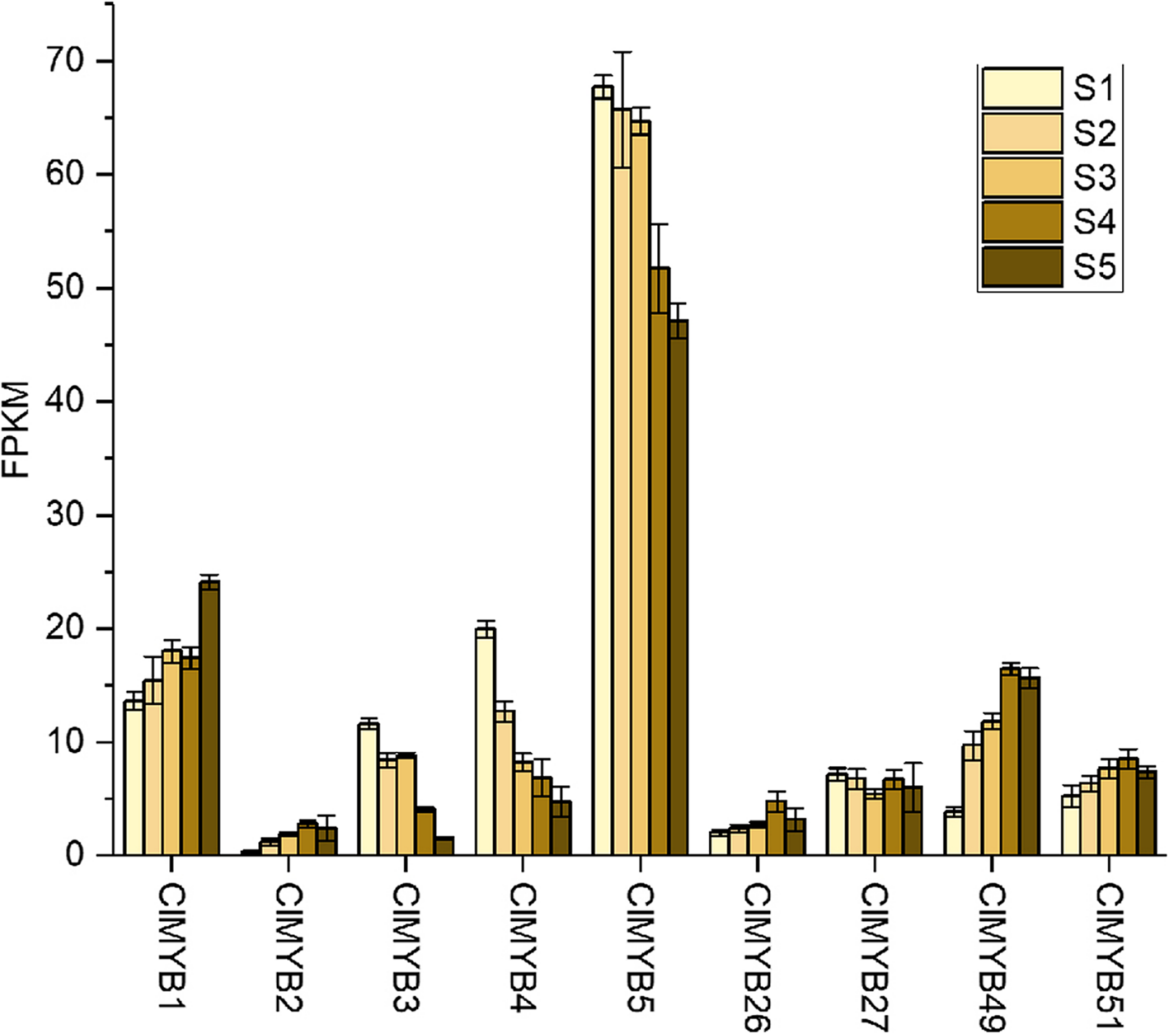
Expression level of selected *ClMYBs* in stem segments with maturation increasing from S1 to S5

The expression profiles of the nine candidates that might involve in regulating secondary wall development in lignified tissues could be divided into three categories: *ClMYB1*, *ClMYB26*, *ClMYB27*, *ClMYB49*, and *ClMYB51* were dominantly expressed in the stem xylem (*ClMYB3* and *ClMYB5*) and (*ClMYB2* and *ClMYB4*) were segregated in sub-clades that exhibited a high expression in the root and female cone, respectively. Since the R2R3-MYBs may act as an activator or repressor to secondary wall development, we followed their expression profile in different stem segments with maturation increasing from S1 to S5 (Fig. 5, Supplementary Table S3). For the xylem specific regulators (*ClMYB1*, *ClMYB26*, *ClMYB49*, and *ClMYB51*), their expression increased congruent with stem maturation; except ClMYB27 that its expression was indifferent in the developmental stem tissues. The expression of *ClMYB3*, *ClMYB4*, and *ClMYB5* in the stems tended to decrease with stem maturation, suggesting they may play negative regulatory roles in wood formation. Interestingly, we found that although ClMYB21 belonged to the woody-preferential subgroup III, its gene expression was not significantly different in the developmental stems. The RNA-Seq data were further verified by qRT-PCR experiments on eight representative samples for 12 selected MYB genes (Supplementary Fig. S3). Correlation coefficients of 0.845–0.956 (*p* < 0.01) indicated that the gene expression patterns obtained from the RNA-Seq data were consistent with the qRT-PCR results.

### Expression patterns of *ClMYBs* involved in compression wood development

**Fig. 6:**
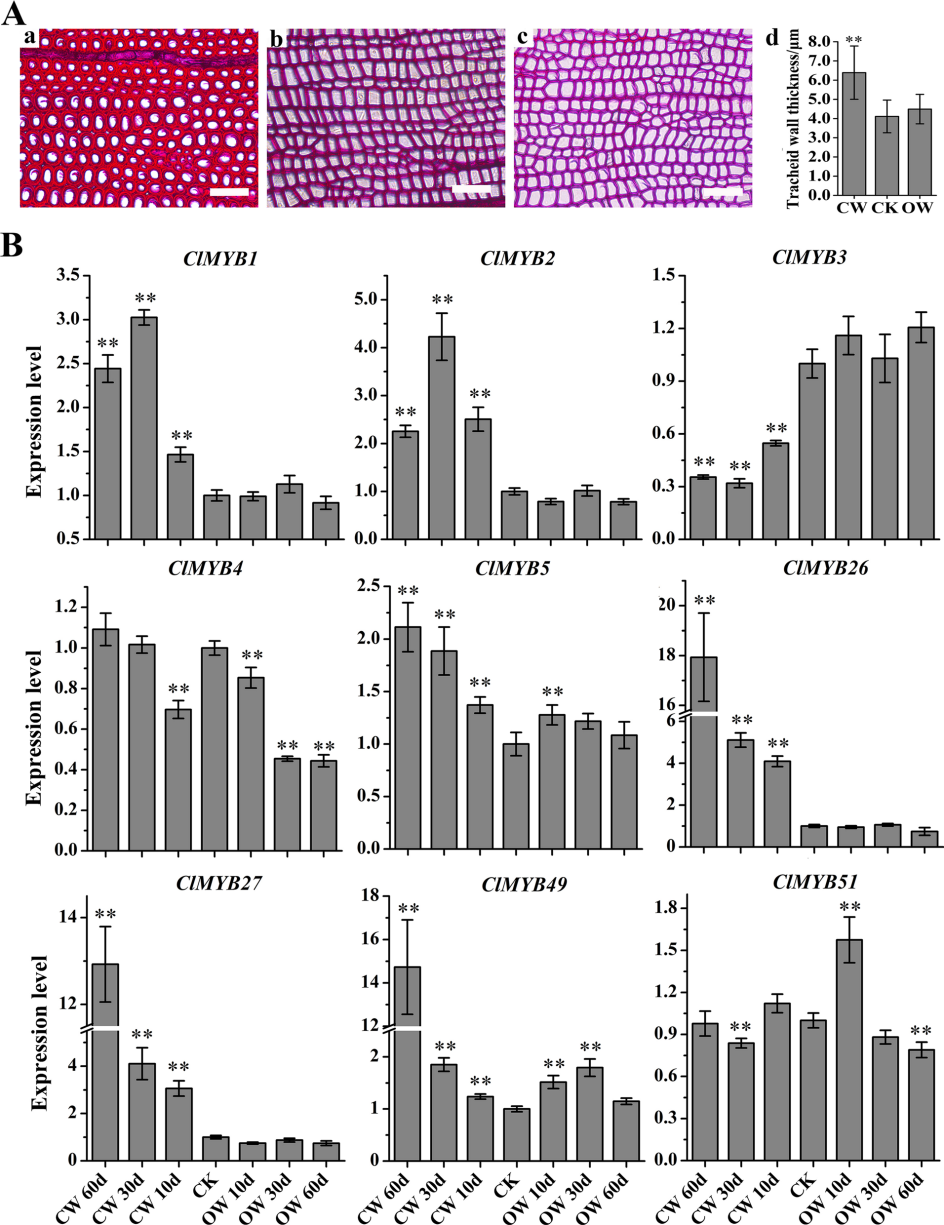
Expression patterns of *ClMYBs* during the compression wood formation. (A) Xylem cross-sections stained with safranin and tracheid wall thickness of different wood styles. a: xylem cross-section of compression wood; b: xylem cross-section of normal wood (CK); c: xylem cross-section of opposite wood. The scale bar is 50 μm. d: tracheid wall thickness of different wood types. (B) The samples were harvested after 10 d, 30 d, 60 d of mechanical bending of *C. lanceolata* plants. Asterisks (**) represented a significant difference between the treatment and control at the 0.01 level. Three biological replicates were used for analysis

Trees develop reaction wood in response to a gravistimulus. In gymnosperms, reaction wood usually develops on the lower side of leaning stems or branches and is referred to as compression wood. Typical features of *C. lanceolate* compression wood could be observed when plants were mechanically bent at a 45° angle from the vertical axis. The tracheid walls of compression wood were significantly thicker than those of normal wood (Fig. 6A, Supplementary Data S2) and contained more lignin but less cellulose [56]. By analysing the expression of ClMYBs encoding genes associated with secondary wall development in the compression wood, we could follow how they were coordinated or reprogrammed that might cause perturbation of anatomical and chemical features of woody tissues in Chinese fir (Fig. 6B, Supplementary Data S3).

Xylem specific ClMYBs generally exhibited increased expression level in compression wood as the bending period prolonged albeit with minor differences. The expression level of *ClMYB26, ClMYB27* and *ClMYB49* exhibited similar expression patterns, as the expression level in the compression wood was significantly greater than that in the upright wood and reached its maximum after 60 days of bending. The expression level of *ClMYB1* in the compression wood was first increased but then decreased with the progression of the bending; also, these levels were significantly higher than those in the upright wood. No significant difference in expression levels between the opposite wood and upright wood, except that *ClMYB49* in opposite wood had shown a significant higher expression level than that in the upright wood. The expression level of *ClMYB51* in both the compression wood and opposite wood fluctuated and did not exhibit a distinct increment in the compression wood as that shown by other xylem specific MYBs.

The responses of the potential negative regulators *ClMYB3*, *ClMYB4*, and *ClMYB5* to bending were also different from each other during compression wood induction. The expression level of *ClMYB3* in the compression wood was significantly lower than that in the control, but the expression level in the opposite wood was not significantly different from that of upright wood. The expression level of *ClMYB4* in the compression wood decreased significantly during the first 10 days and then returned to values similar to that in the upright wood, while the corresponding values of the opposite wood were significantly lower than those of the control and were 0.45 and 0.44 at 30 and 60 days after bending, respectively. Additionally, the expression of *ClMYB5* in the compression wood gradually increased, reaching a maximum (2.11) after 60 days of bending. Only after ten days of bending, the expression of this gene in the opposite wood was significantly different from that in the control.

### Subcellular localization and transcriptional activity analysis of ClMYB1

**Fig. 7:**
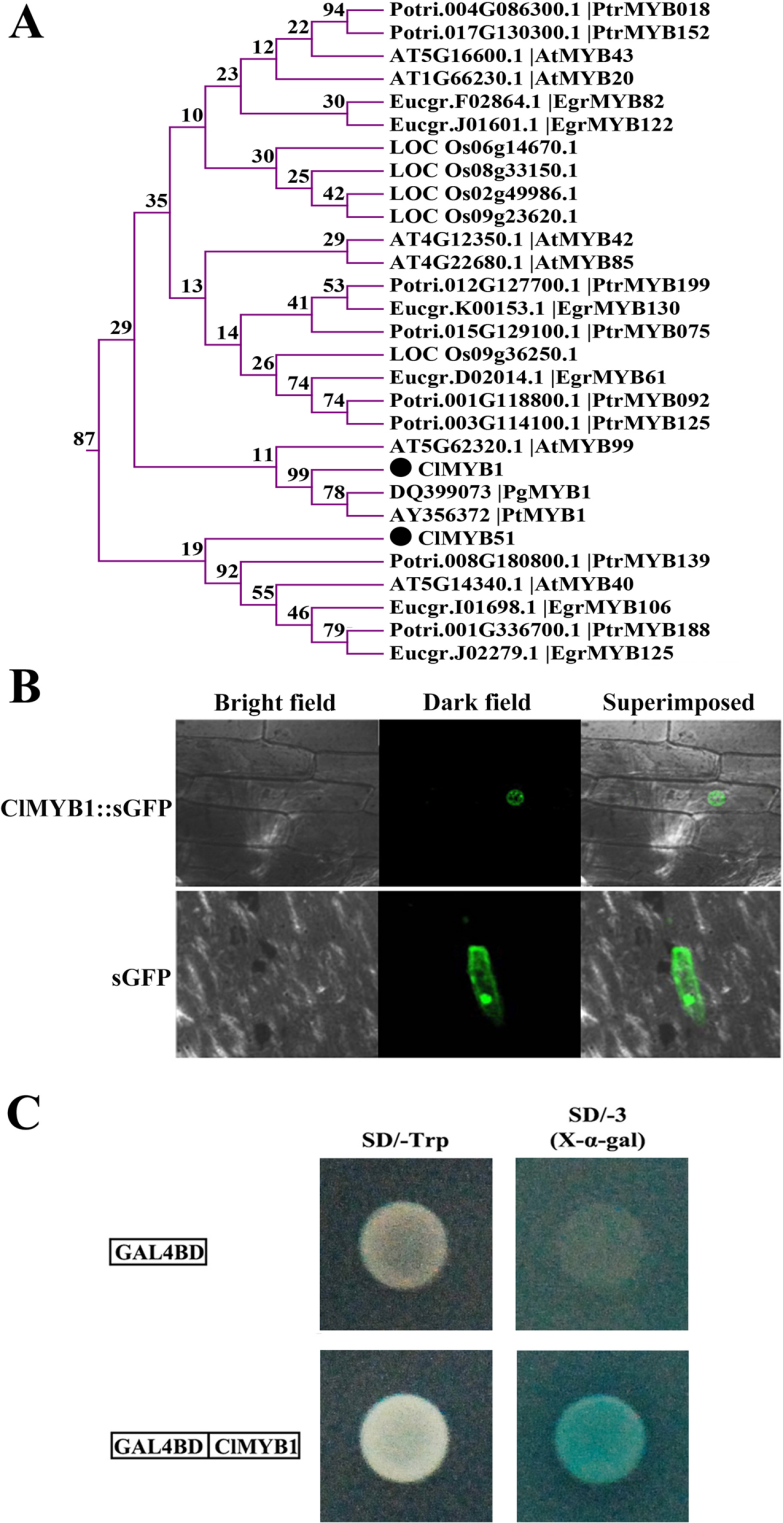
Subcellular localization and transcriptional activity analysis of ClMYB1. (**A**) Phylogenetic tree of the MYB subgroup including ClMYB1. (**B**) Subcellular localization of ClMYB1 in onion epidermal cells. (**C**) Yeast one-hybrid assay. SD/−Trp, yeast SD medium lacking tryptophan. SD/− 3, yeast SD medium lacking tryptophan, histidine, and Adenine

The phylogenetic and gene expression analysis suggested that the function of the xylem specific ClMYBs related to the regulation of secondary wall development might be conserved in *C. lanceolata*. To verify the protein function, we examined the functionality of *ClMYB1* (GenBank accession number: JQ904045) by subcellular localization and overexpression in tobacco plant. *ClMYB1* was highly homologous to *PtMYB1* and *PtrMYB152* (Fig. 7A) and was expressed predominantly in the developing stem, suggesting that this gene participated in secondary wall development in *C. lanceolata*. The subcellular localization showed that the control GFP was distributed throughout the whole epidermal cell, while the ClMYB1:GFP fusion protein accumulated exclusively in the nucleus (Fig. 7B). The results indicated that ClMYB1 is localised in the nucleus, which is consistent with its function as a transcription factor. As shown in Fig. 7C, the yeast transformants harboring the negative control plasmid pGBKT7 and the recombinant plasmid pGBKT7-ClMYB1 were both able to grow on SD/−Trp media, but only the yeast containing the recombinant plasmid grew well on the selection media and induced the expression of the α-galactosidase reporter gene, indicating that ClMYB1 is a transcriptional activator. Taken together, these results indicated that ClMYB1 functions as a transcriptional activator in the nucleus.

### Ectopic expression of *ClMYB1* in *N. benthamiana*

**Fig. 8:**
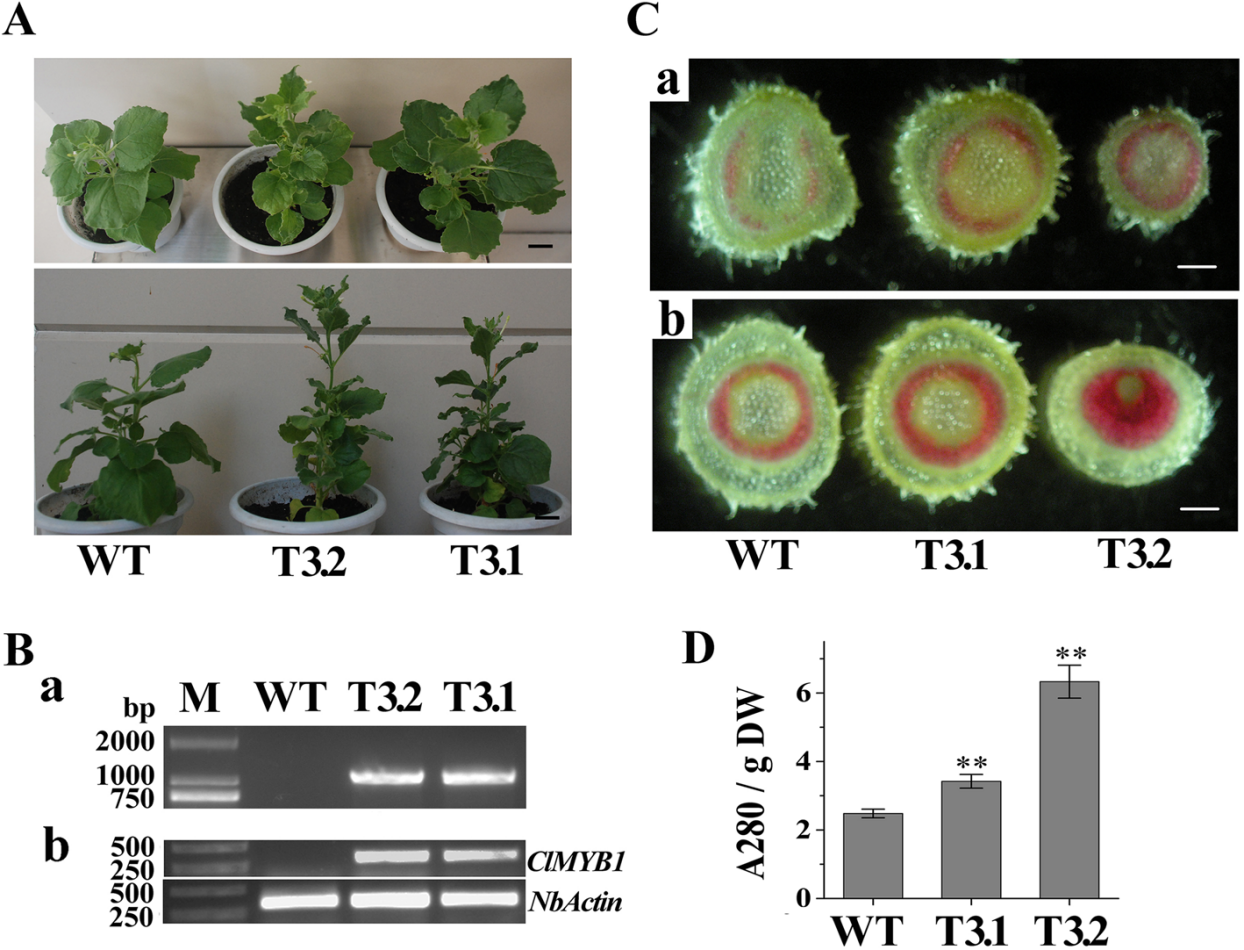
Phenotypic modification of *ClMYB1* over-expression in tobacco. (A) Morphological comparison of *ClMYB1* overexpressing lines (T3.1 and T3.2) and wild type (WT). The scale bar is 1.5 cm. (B) PCR analysis of target sequence in transformed lines (a) and expression analysis of *ClMYB1* gene in transformed lines by semi-qRT-PCR (b). (C) Stem cross-sections of ClMYB1 overexpressing lines and WT. The sections were prepared from the middle (a) and bottom (b) parts of stems. The scale bar is 1.0 mm. (D) Lignin content comparison of *ClMYB1* overexpressing lines and WT. DW: dry weight

To investigate its regulatory functions in secondary vascular development, *ClMYB1* gene was successfully transformed into *N. benthamiana*. Eight putative transgenic lines were obtained, and two *ClMYB1*-overexpressed lines were selected for in-depth characterization. PCR revealed that *ClMYB1* had been successfully introduced into the *N. benthamiana* genome and was transcribed in transformed lines (Fig. 8B). Compared with the wild-type (WT), the transgenic lines exhibited smaller leaves with an undulate margin and a smaller angle between the petiole of the upper leaves and the main stem (Fig. 8A). Cross-sections of the top, middle and bottom parts of the stems were prepared for phloroglucinol-HCl staining. No significant difference was observed from staining results between the upper stem segments of the transgenic lines and WT, but darker staining was observed in the xylem regions of the middle and bottom stem segments from transgenic lines (Fig. 8C). A subsequent assay showed that the lignin content of the stems from transgenic lines was significantly higher than that of wild type (Fig. 8D, Supplementary Data S4). These results indicated that *ClMYB1*-overexpression could promote lignin deposition in the xylem regions of stems.

## Discussion

Large genome size and limited gene information severely hinder the study of molecular mechanisms on critical biological processes related to organ development and stress resistance in many valuable coniferous timber species. Using an advanced sequencing technology, 52 R2R3-MYB encoding genes with complete ORFs were obtained from the full-length transcriptomic libraries of *C. lanceolata*. Although not as many as in *Arabidopsis* (126 *AtMYBs*) and *Populus* (187 *PtrMYBs*), the number of identified ClMYBs were distinctly greater than that previously reported in the conifers such as *P. taeda* and *P. glauca* [6, 55]. This work effectively updated and improved the collection of full-length gymnosperms specific R2R3-MYB transcription factors related to stem development.

### Evolutionary relationship of R2R3-MYBs from *C. lanceolata*

R2R3-MYBs are crucial in the regulation of plant development [8]. The number of R2R3-MYBs increased gradually in basal land plants and eventually expanded rapidly after the emergence of seed plants (spermatophyte) [21]. Depending on the mechanism of gene duplication, some duplicated genes may undergo sub- or neo-functionalization that implicated in the diversification of gene function [57]. The comparative analysis of gene mapping on the spruce genome with those of monocots and *A. thaliana* suggests that the number of gene duplication shared by angiosperm and gymnosperm is greater than that of gymnosperm specific gene expansion [58]. Similarly, the R2R3-MYBs from *C. lanceolata* belonged to most subgroups shared by monocot and dicots, indicating that these subgroups might share the common ancestor. Only a small number of subgroups were specific to gymnosperm lineage. There is an excellent scope for characterization of the candidate genes to hypothesize new functions related to the coniferous developmental growth. The dominant expression of gene encoding ClMYB24 of subgroup S13b in xylem tissues, inferred it a potential regulator of stem development in gymnosperms.

It has been shown that the divergence between monocot and dicotyledonous species, which accompanied by whole-genome duplications [59], resulted in the speciation of subgroups limited to monocots and/or dicotyledonous lineages [9, 12]. Out of the five woody-preferential subgroups identified by Soler et al., (2015) [12], only one of them harboured the ClMYB21 from *C. lanceolata*. In contrast, the woody-expanded subgroups that were typically enriched in R2R3-MYBs of woody species, but also carrying one or two sequences from *A. thaliana* or *O. sativa*, contained a high number of sequences from *C. lanceolata.* In particular, the woody-expanded subgroup SAtM5 contained up to six ClMYBs, with their expression being variably expressed in different part of organs. There are two possible reasons for the absence of ClMYBs in majority of the woody-preferential subgroup: 1) the corresponding transcripts were not detected in the present transcriptomic libraries; 2) most of the woody-preferential subgroups have emerged after the gymnosperm/angiosperm split. The overrepresentation of ClMYBs in the woody-expanded subgroups suggested that the subgroup appears predating the divergence of angiosperm-gymnosperm and underwent asymmetric gene duplication and diversification in woody species [20].

### Involvement of ClMYBs in the regulation of secondary wall development

The ClMYBs resided in some of the *A. thaliana* secondary cell wall-related subgroups based on the phylogeny analysis. We also observed their expression in different organ and developmental stem, as well as compression wood to justify their involvement in secondary wall development. It was hypothesized that the functional role of the xylem specific ClMYB1/26/49/51 might be directly involved in the upregulation of secondary wall development in the stem of *C. lanceolata*. Their categorisation with those of *A. thaliana* and woody species was supported by a high bootstrap value. ClMYB1 and ClMYB51 were clustered in subgroup SAtm85 (bootstrap value 83%), which included several MYBs related to secondary wall development, such as AtMYB85, PtrMYB152, and PtMYB1. AtMYB85, a target gene of AtMYB46/83, regulate lignin biosynthesis in *A. thaliana* [39] and is highly expressed in the xylem of inflorescence stems and roots [60]. PtrMYB152 is a transcriptional activator specific for lignin biosynthesis in *P. trichocarpa* and is expressed mainly in lignified tissues [61]. MYB1 of *P. taeda* activates the transcription of genes encoding lignin biosynthetic enzymes by binding with AC elements and is mainly expressed in developing xylem [19]. *ClMYB1* and *ClMYB51* were expressed mainly in the developing xylem of the stem, and their expression levels in the stem increased with lignification. In response to the bending treatment, the expression of *ClMYB1* was upregulated, corresponding to the lignin content in the compression wood. To date, no available genetic transformation system has been established for *C. lanceolata*, so the biological functions of most candidates involved in the wood formation of this species remain mostly unexplored. In this study, we selected ClMYB1 for further functional characterization, based on subcellular localization and transcriptional activity analysis. Functional analysis by overexpression of *ClMYB1* in *N. banthamiana* indicated that it could increase lignin deposition in the stems. The results indicated the retention of function between ClMYB1, AtMYB85 and PtrMYB152, although the conservation of their activators and downstream targets remains to be studied further.

The expression patterns of *ClMYB49* in different organs, tissues and during the formation of compression wood were essentially consistent with those of *ClMYB1*, indicating that they may also be involved in the regulation of secondary wall development in *C. lanceolata*. The sequences of ClMYB49 were segregated in subgroup SAtm46 together with PtMYB4 and AtMYB46/83 (bootstrap value 91%). AtMYB46/83 activated the transcriptional activity of secondary wall biosynthesis and was directly controlled by the NAC domain-containing SND1 master regulator in *A. thaliana* [37, 38, 62]. *PtMYB4* was expressed mainly in the xylem and its protein can bind to AC elements. The overexpression of this gene in tobacco lead to an increase in lignin content [18]. ClMYB26 was clustered in the subgroup SAtM103 with AtMYB103, PtrMYB010, and PtrMYB128 (bootstrap value 97%). Intriguingly, AtMYB103 influenced syringyl-lignin synthesis by regulating the expression of ferulate-5-hydroxylase (F5H) gene [39, 50]. S-lignin is typically enriched in the secondary wall of dicots, while gymnosperms have only a small amount of S-lignin [63]. The regulatory process of ClMYB26 will be subjected to future investigation to identify the downstream targets and the influence on lignin composition. The specificity of the regulatory mechanism of ClMYB26 and AtMYB103 towards the biosynthesis of monolignol would clarify whether divergence of function has occurred after the angiosperm/gymnosperm split.

### Repressive role of ClMYBs in subgroup S4

The expression of *ClMYB3*, *ClMYB4*, and *ClMYB5* in the stems decreased with increasing lignification, and these genes clustered with AtMYB4 and EgrMYB1 in subgroup 4. Both AtMYB4 and EgrMYB1 are negative regulators of lignin biosynthesis. *EgrMYB1* is expressed predominantly in the secondary xylem of root and stem [64]. Therefore, ClMYB3/4/5 could be the potential regulators of *C. lanceolata* wood formation by negatively regulating lignin biosynthesis. Unlike the xylem specific ClMYBs, ClMYB3/4/5 received only 46% support for their classification in subgroup S4. The probable reason of this might be the diversity of C-terminal region in the orthologs. Compared with angiosperm, subgroup S4 was asymmetrically expanded in gymnosperm species such as *P. glauca* and *P. taeda* [55]. ClMYB3/4/5 carried a unique motif with core sequence of (E/I)LIETADFFQ. The similar motif was also detected when aligning with the *P. glauca* orthologs (Pg5/10/13). Still, none were found in the orthologs of *O. sativa*, *E. grandis*, and *P. trichocarpa*, suggesting this motif was specific to gymnosperm lineage. Since the role of this unique motif is unknown, the regulatory process involving ClMYB3/4/5 may en-route differently, though they share the repressive EAR motif responding to external stimuli and hormonal signals [65] with angiosperms. Moreover, in response to the bending treatment, *ClMYB3* and *ClMYB5* were down- and upregulated respectively with the development of compression wood. These all observations suggested that they might be recruited differently in the regulatory process, hence targeting different downstream targets, and playing different roles in wood formation responding to defence and stress stimulation.

## Conclusions

A total of 52 R2R3-MYB encoding genes with complete ORFs were obtained from the full-length transcriptomic libraries of *C. lanceolata* developmental stem. Though they were mostly segregated in the subgroups harbouring those from monocot and dicot species, a minor portion of the subgroups strictly carried members of gymnosperm origin. The asymmetric distribution of the ClMYBs among woody-preferential and woody-expanded subgroups indicated that woody species of angiosperm and gymnosperm underwent gene expansion in the woody-expanded subgroups. Nine candidates shared the same subgroups with *A. thaliana*, from which ClMYB1/2/5/26/27/49 may be directly involved in the upregulation of cell wall development. Functional analysis of ClMYB1 in *N. banthamiana* demonstrated its positive regulation in lignification. Our findings will contribute to a greater understanding of the evolutionary relationship of ClMYBs with those from the angiosperms. The information obtained will provide important clues for the selection of candidates for use in improving the wood quality of *C. lanceolata*.

## Supplementary Information

**Additional file 1: Figure S1.** Cross-sections stained with Phloroglucinol-HCl for the different lignification stems of *C. lanceolata*. Five stem segments from the top to bottom of a one-year-old branch were collected and marked sequentially S1, S2, S3, S4, and S5.

**Additional file 2: Figure S2.** Phylogenetic tree of 629 R2R3-MYB proteins from *Cunninghamia lanceolata* (Cl), *Eucalyptus grandis* (Egr), *Populus trichocarpa* (Ptr), *Arabidopsis thaliana* (At), *Oryza sativa* (Os), *Pinus taeda* (Pt), and *Picea glauca* (Pg). All proteins were divided into 47 subgroups.

**Additional file 3: Figure S3.** Expression profiles of 12 *ClMYB* genes in eight organs and tissues using qRT-PCR. Data were normalised to *ClActin* gene. The expression level in S1 was used as a reference and was set at 1. Error bars indicate standard deviation of three biological replicates. The corresponding FPKM values were listed. The *r* represented the correlation coefficient between qRT-PCR and RNA-Seq data, and the *p* was the *P*-value. L: leaves, S1-5: stem segments from top to bottom, X: xylem, B: bark.

**Additional file 4: Table S1.** Primers used in the analysis of qRT-PCR, subcellular localization, yeast hybridization, overexpression, and semi-qRT-PCR.

**Additional file 5: Table S2.** Physicochemical properties and protein-coding sequences of ClMYB transcription factors.

**Additional file 6: Table S3.** FPKM values of 52 *ClMYB* genes in different organs or tissues.

**Additional file 7: Data S1.** Descriptions and sequence logo plots of motifs in ClMYB proteins.

**Additional file 8: Data S2.** Tracheid wall thickness of different samples.

**Additional file 9: Data S3.** Quantification Cq results of 9 *ClMYB* genes in qRT-PCR.

**Additional file 10: Data S4.** Lignin contents of stems from transgenic lines and wide-type.

**Additional file 11: Electrophoretogram 1.** *ClMYB1* PCR detection of genomic DNA in transgenic *N. benthamiana*. M: DNA marker (DL2000, TaKARa), WT: wide-type, T3.1 and T3.2: two T3 generation lines.

**Additional file 12: Electrophoretogram 2.** *ClMYB1* transcripts detection by semi-quantitative RT-PCR in transgenic *N. benthamiana*. A, Transcript detections of *ClActin* (lane 2–7) and *ClUBC* (lane 8–13). B, Transcript detections of *ClGAPDH* (lane 2–7) and *ClMYB1* (lane 8–13). M: DNA marker (DL2000, TaKaRa), WT: wide-type, T3.1 and T3.2: two T3 generation lines. T2.1, T2.2 and T2.3: three T2 generation lines.

**Additional file 13.**

## Data Availability

The raw sequence data reported in this paper have been deposited in the Genome Sequence Archive [66] in BIG Data Center [67], Beijing Institute of Genomics (BIG), Chinese Academy of Sciences, under accession numbers CRA003755 that are publicly accessible at http://bigd.big.ac.cn/gsa. The datasets supporting the conclusions of this article are included within the article and its supplemental files.

## Abbreviations

B: bark
CK: control plant
CW: compression wood
EST: express sequence tag
FC: female cone
L: needle-leaves
MC: male cone
OW: opposite wood
SMRT: Single-Molecule Real-Time
RT: root
X: xylem
VNS: VND, NST and SMB homolog
