# Supplementary material for: Full-length transcriptomic identification of R2R3-MYB family genes related to secondary cell wall development in Cunninghamia lanceolata (Chinese fir)
Source: BMC Plant Biol. 2021 Dec 8;21:581. doi: 10.1186/s12870-021-03322-w (PMC8653563; doi:10.1186/s12870-021-03322-w)
Supplement: Supplementary file 1 — Additional file 1: Figure S1. Cross-sections stained with Phloroglucinol-HCl for the different lignification stems of C. lanceolata. Five stem segments from the top to bottom of a one-year-old branch were collected and marked sequentially S1, S2, S3, S4, and S5. [file 12870_2021_3322_MOESM1_ESM.pdf]

Full-length transcriptomic identification of R2R3-MYB family genes related to secondary cell wall development in *Cunninghamia lanceolata* (Chinese fir)

Hebi Zhuang[1§], Sun-Li Chong[1§], Borah Priyanka[1], Xiao Han[1], Erpei Lin[1], ZaiKang Tong[1], HuaHong Huang[1]

[1]State key Laboratory of Subtropical Silviculture, Zhejiang A/F University, Linäan, Hangzhou 311300, China
[§]These authors contributed equally to this work

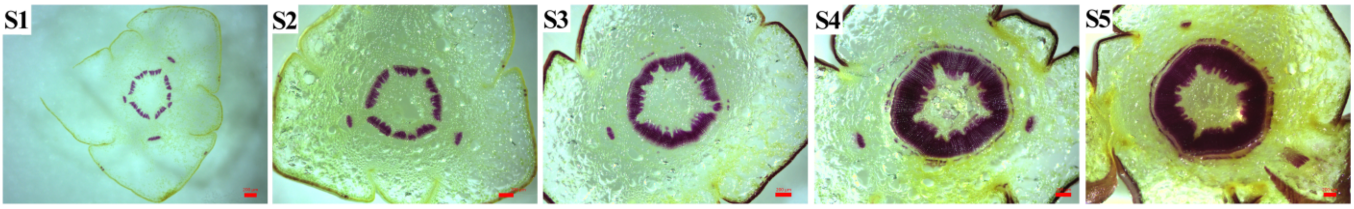

Figure S1. Cross-sections stained with Phloroglucinol-HCl for the different lignification stems of *C. lanceolata*. Five stem segments from the top to bottom of a one-year-old branch were collected and marked sequentially S1, S2, S3, S4, and S5.
