# Supplementary material for: Full-length transcriptomic identification of R2R3-MYB family genes related to secondary cell wall development in Cunninghamia lanceolata (Chinese fir)
Source: BMC Plant Biol. 2021 Dec 8;21:581. doi: 10.1186/s12870-021-03322-w (PMC8653563; doi:10.1186/s12870-021-03322-w)
Supplement: Supplementary file 2 — Additional file 2: Figure S2. Phylogenetic tree of 629 R2R3-MYB proteins from Cunninghamia lanceolata (Cl), Eucalyptus grandis (Egr), Populus trichocarpa (Ptr), Arabidopsis thaliana (At), Oryza sativa (Os), Pinus taeda (Pt), and Picea glauca (Pg). All proteins were divided into 47 subgroups. [file 12870_2021_3322_MOESM2_ESM.pdf]

Full-length transcriptomic identification of R2R3-MYB family genes related to secondary cell wall development in *Cunninghamia lanceolata* (Chinese fir)

Hebi Zhuang[1§], Sun-Li Chong[1§], Borah Priyanka[1], Xiao Han[1], Erpei Lin[1], ZaiKang Tong[1], HuaHong Huang[1]

[1]State Key Laboratory of Subtropical Silviculture, Zhejiang A&F University, Lin'an, Hangzhou 311300, China
[§]These authors contributed equally to this work

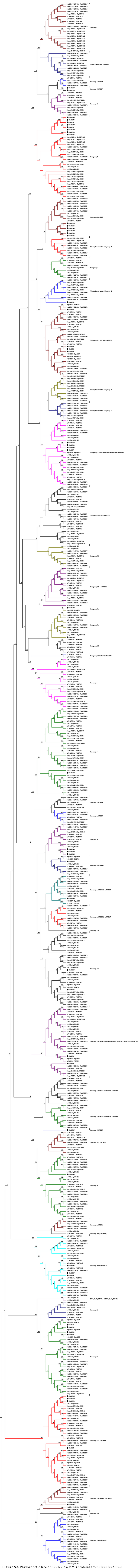

Figure S2. Phylogenetic tree of 629 R2R3-MYB proteins from *Cunninghamia lanceolata* (Cl), *Eucalyptus grandis* (Egr), *Populus trichocarpa* (Ptr), *Arabidopsis thaliana* (At), *Oryza sativa* (Os), *Pinus taeda* (Pt), and *Picea glauca* (Pg). All proteins were divided into 47 subgroups
