# Supplementary material for: Full-length transcriptomic identification of R2R3-MYB family genes related to secondary cell wall development in Cunninghamia lanceolata (Chinese fir)
Source: BMC Plant Biol. 2021 Dec 8;21:581. doi: 10.1186/s12870-021-03322-w (PMC8653563; doi:10.1186/s12870-021-03322-w)
Supplement: Supplementary file 3 — Additional file 3: Figure S3. Expression profiles of 12 ClMYB genes in eight organs and tissues using qRT-PCR. Data were normalised to ClActin gene. The expression level in S1 was used as a reference and was set at 1. Error bars indicate standard deviation of three biological replicates. The corresponding FPKM values were listed. The r represented the correlation coefficient between qRT-PCR and RNA-Seq data, and the p was the P-value. L: leaves, S1-5: stem segments from top to bottom, X: xylem, B: bark. [file 12870_2021_3322_MOESM3_ESM.pdf]

Full-length transcriptomic identification of R2R3-MYB family genes related to secondary cell wall development in *Cunninghamia lanceolata* (Chinese fir)

Hebi Zhuang[1§], Sun-Li Chong[1§], Borah Priyanka[1], Xiao Han[1], Erpei Lin[1], ZaiKang Tong[1], HuaHong Huang[1]

[1]State Key Laboratory of Subtropical Silviculture, Zhejiang A&F University, Lin'an, Hangzhou 311300, China
[§]These authors contributed equally to this work

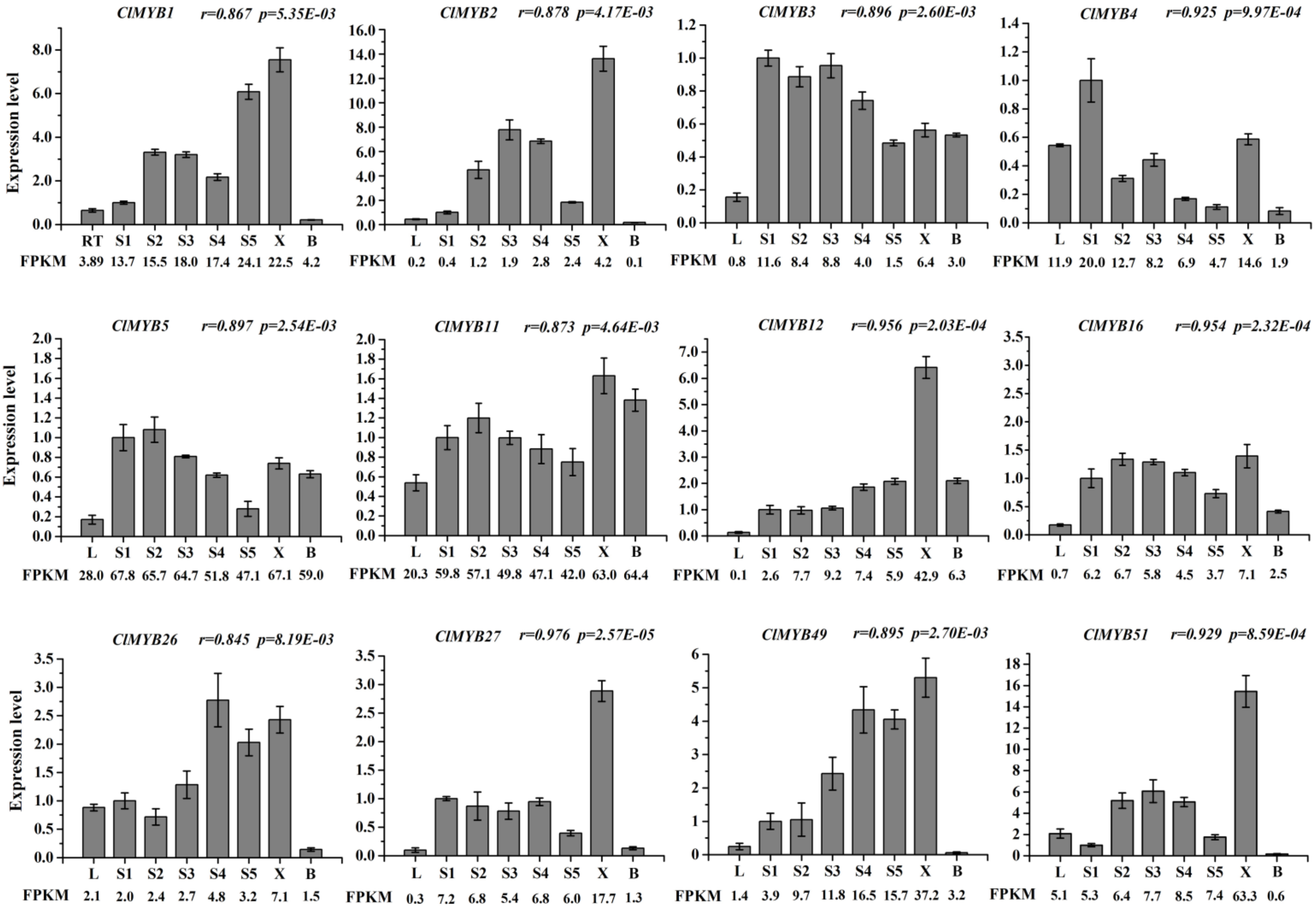

Figure S1. Expression profiles of 12 *ClMYB* genes in eight organs and tissues using qRT-PCR. Data were normalised to *ClActin* gene. The expression level in S1 was used as a reference and was set at 1. Error bars indicate standard deviation of three biological replicates. The corresponding FPKM values were listed. The *r* represented the correlation coefficient between qRT-PCR and RNA-Seq data, and the *p* was the P-value. L:leaves, S1-5: stem segments from top to bottom, X: xylem, B: bark
