# Supplementary material for: Full-length transcriptomic identification of R2R3-MYB family genes related to secondary cell wall development in Cunninghamia lanceolata (Chinese fir)
Source: BMC Plant Biol. 2021 Dec 8;21:581. doi: 10.1186/s12870-021-03322-w (PMC8653563; doi:10.1186/s12870-021-03322-w)
Supplement: Supplementary file 4 — Additional file 4: Table S1. Primers used in the analysis of qRT-PCR, subcellular localization, yeast hybridization, overexpression, and semi-qRT-PCR. [file 12870_2021_3322_MOESM4_ESM.docx]

**Full-length transcriptomic identification of R2R3-MYB family genes related to secondary cell wall development in *Cunninghamia lanceolata* (Chinese fir)**

Hebi Zhuang^1§^, Sun-Li Chong^1§^, Borah Priyanka^1^, Xiao Han^1^, Erpei Lin^1^, Zaikang Tong^1^, Huahong Huang^1*^

^1^State Key Laboratory of Subtropical Silviculture, Zhejiang A&F University, Lin'an, Hangzhou 311300, China

^§^These authors contributed equally to this work.

Table S1. Primers used in the analysis of qRT-PCR, subcellular localization, yeast hybridization, overexpression, and semi-qRT-PCR.

| Function | Gene | Forward primer (5’ - 3’) | Reverse primer (5’ - 3’) |
| --- | --- | --- | --- |
| qRT-PCR | *ClActin* | GTACTGCTTGTAGGTGGAGTTGC | TAGAATACCAAGAACAGCACCAC |
|  | *ClMYB1* | GCGATTGGCTTCATTGCTTGAG | TACAGAGGAACACACGAGCGATG |
|  | *ClMYB2* | GAATGAGCAACATAAGGGAGGAG | TCTTAGGTTCCAATGTAGCCAGC |
|  | *ClMYB3* | CAGAACTCCGAAGTAACCAGCTC | GGTGGAACAAGGCAAACTGATAC |
|  | *ClMYB4* | AGGTCTGGCTTATGATTACAGGC | CAGAGGCAACAAATGCTTATCTAC |
|  | *ClMYB5* | GGAGTATGGATTTCCAAACCCAG | CGATCAACCCAAGAGCTTTCATC |
|  | *ClMYB11* | GCTCTGTGTTGTCTGCCATTGTAG | CCCTTTATTCTCTCACGCTCTCTC |
|  | *ClMYB12* | AGCAAGCAGGACTTTTGAGATGTG | CATTATCTGTTCTTCCAGGCAGGC |
|  | *ClMYB16* | GACTGTAAACGGGCATTTCATAG | GCATCTCTATTCACAGACACAGC |
|  | *ClMYB24* | GGACACATTCTCAAGCTGAAG | GTACGAGGATAGCTGTTCAGTG |
|  | *ClMYB25* | TGGATCTCAGACTGTATTGTCAG | GTTGAAAGCTGAATGTCATGCAC |
|  | *ClMYB26* | GGAATAGCAGCAGCAACATAGAG | ATGTCCAGAAGGCTTGGTTTCAG |
|  | *ClMYB42* | CGCTGTGGAATATTGACATGGAG | TAGTCGTGGTGCATTTGTGATAC |
|  | *ClMYB49* | GCTGATTGTAAGGGTGATGACAG | CTCCTCCTAATGAATTACCTGTG |
|  | *ClMYB51* | CAGCCATCAAGTGACTGTGCCTC | CCGAAGGCTGGCTAGACGAAGAG |
| Subcellular localization | *ClMYB1* | CGGGGTACCACTATGGGAAGGCAGCCGTGCT | GCTCTAGACATTTCCTCAAGCAATGAAGCCA |
| Yeast hybridization | *ClMYB1* | ATGGGAAGGCAGCCGTGCTGTG | CATTTCCTCAAGCAATGAAGC |
| Overexpression | *ClMYB1* | CGGGGTACCCTCGCATACTATGGGAAGGCAGC | GCTCTAGATACAGAGGAACACACGAGCGATG |
| Semi-qRT-PCR | *ClMYB1* | TGCTGCTTCTGTGGATAACAATG | TACAGAGGAACACACGAGCGATG |
|  | *NbActin* | CTAGAGACTTCAAAGACCAGCTC | ATAGAGCCTCCTATCCAGACACT |
