# Supplementary figures and images for: Full-length transcriptomic identification of R2R3-MYB family genes related to secondary cell wall development in Cunninghamia lanceolata (Chinese fir)
Source: BMC Plant Biol. 2021 Dec 8;21:581. doi: 10.1186/s12870-021-03322-w (PMC8653563; doi:10.1186/s12870-021-03322-w)

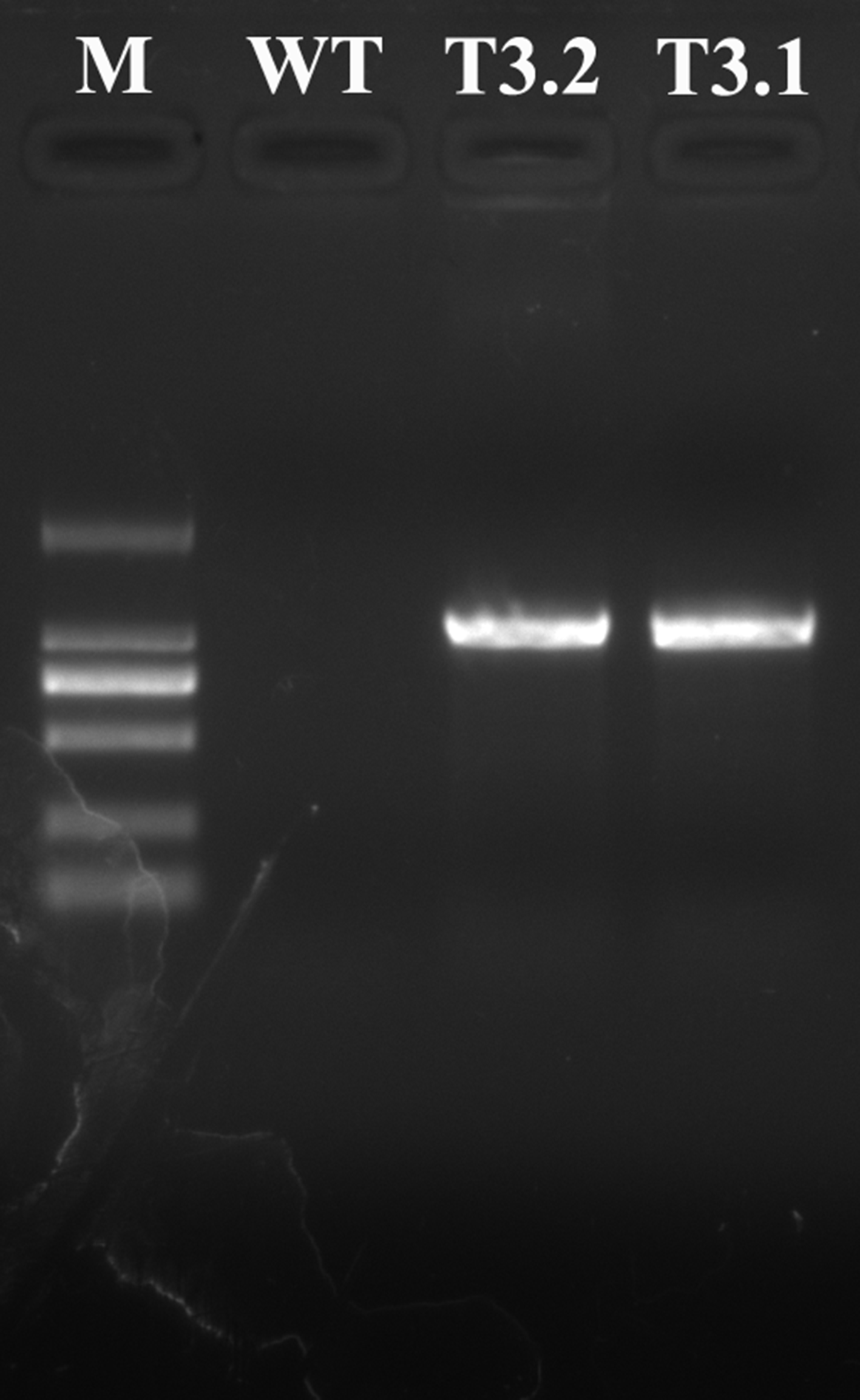

Supplement: Supplementary file 11 — Additional file 11: Electrophoretogram 1. ClMYB1 PCR detection of genomic DNA in transgenic N. benthamiana. M: DNA marker (DL2000, TaKARa), WT: wide-type, T3.1 and T3.2: two T3 generation lines. [file 12870_2021_3322_MOESM11_ESM.tif]

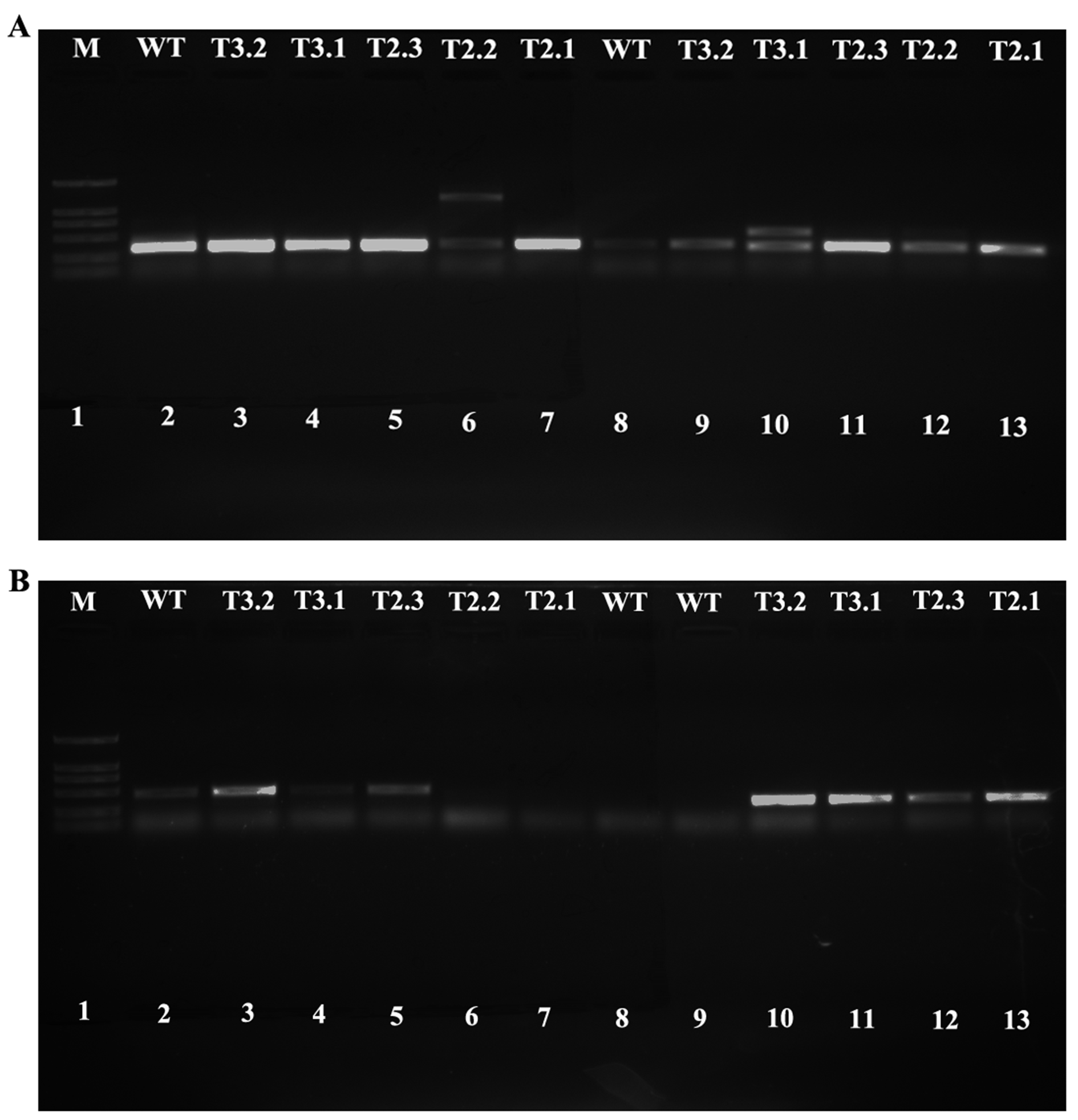

Supplement: Supplementary file 12 — Additional file 12: Electrophoretogram 2. ClMYB1 transcripts detection by semi-quantitative RT-PCR in transgenic N. benthamiana. A, Transcript detections of ClActin (lane 2–7) and ClUBC (lane 8–13). B, Transcript detections of ClGAPDH (lane 2–7) and ClMYB1 (lane 8–13). M: DNA marker (DL2000, TaKaRa), WT: wide-type, T3.1 and T3.2: two T3 generation lines. T2.1, T2.2 and T2.3: three T2 generation lines. [file 12870_2021_3322_MOESM12_ESM.tif]
