# Supplementary material for: Full-length transcriptomic identification of R2R3-MYB family genes related to secondary cell wall development in Cunninghamia lanceolata (Chinese fir)
Source: BMC Plant Biol. 2021 Dec 8;21:581. doi: 10.1186/s12870-021-03322-w (PMC8653563; doi:10.1186/s12870-021-03322-w)
Supplement: Supplementary file 13 — Additional file 13. [file 12870_2021_3322_MOESM13_ESM.docx]

Full-length transcriptomic identification of R2R3-MYB family genes related to secondary cell wall development in *Cunninghamia lanceolata* (Chinese fir)

Hebi Zhuang^1§^, Sun-Li Chong^1§^, Borah Priyanka^1^, Xiao Han^1^, Erpei Lin^1^, Zaikang Tong^1^, Huahong Huang^1*^

^1^State Key Laboratory of Subtropical Silviculture, Zhejiang A&F University, Lin'an, Hangzhou 311300, China

^§^These authors contributed equally to this work.

^
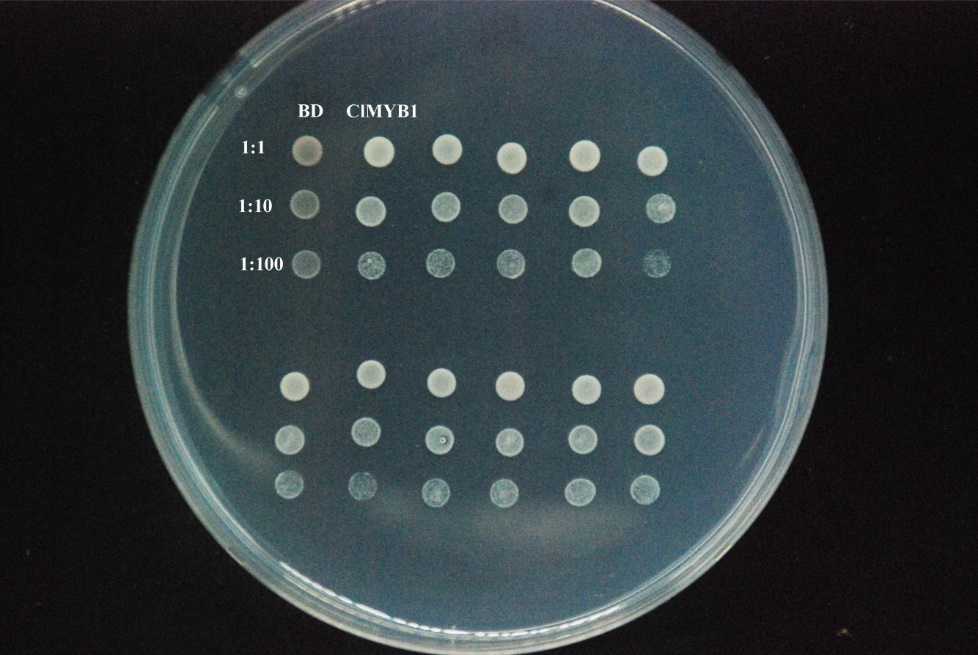
^

**SD/-Trp**

^
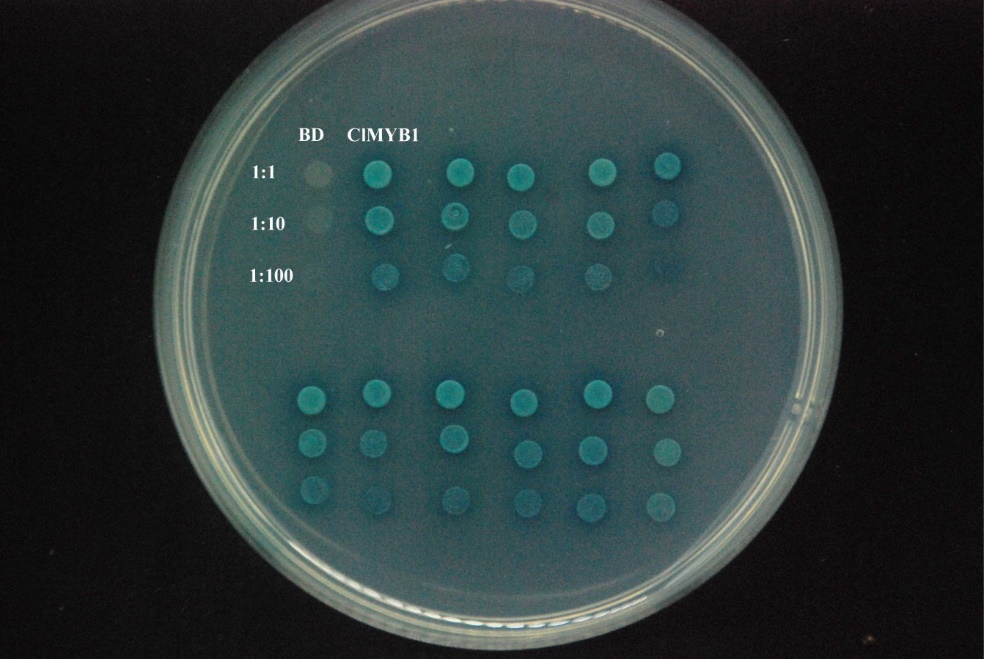
^

**SD/-3**

# Image 1. The original gel plate photo of Figure 7c in the manuscript

The gel plate photo illustrated transcriptional activation assay of *ClMYB1*. The yeast strain harboring empty vector (BD) and *ClMYB1* were grown in SD medium lacking tryptophan (SD/-Trp) and three amino acid elements: tryptophan, histidine, and Adenine (SD/-3); to test the ability of *ClMYB1* on self-activation. Those spots diluted in 1:1 was cropped and used in Figure 7c in the manuscript (red rectangle box).


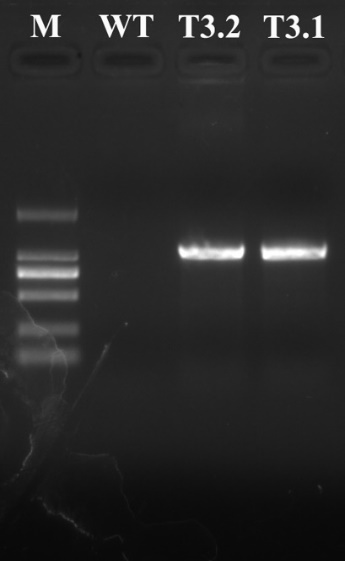


# Image 2. The original gel photo of Figure 8B(a) in the manuscript.

The gel photo contained PCR band of *ClMYB1* amplified from the genomic DNA in transgenic *N. benthamiana* and wild type plant. M: DNA marker (DL2000, TaKARa), WT: wide-type, T3.1 and T3.2: two independent T3 generation lines.

*ClUBC*

*ClActin*





*ClMYB1*

*ClGAPDH*

# Image 3. The original gel photo of Figure 8B(b) in the manuscript.

The gel photo contained *ClMYB1* (lane 8-13) transcripts detection by semi-quantitative RT-PCR in transgenic *N. benthamiana* and wild type plant with *ClActin* (lane 2-7) was used as reference gene. The portion showing amplified bands of *ClMYB1* and *ClActin* in WT, T3.1 and T.32 (red rectangle box) were cropped and used in Figure 8B(b). M: DNA marker (DL2000, TaKaRa), WT: wide-type, T3.1 and T3.2: two independent T3 generation lines. T2.1, T2.2 and T2.3: three independent T2 generation lines.
